# Carnivorous Plant Biology: From Gene to Traps

**DOI:** 10.3390/ijms242216179

**Published:** 2023-11-10

**Authors:** Bartosz J. Płachno

**Affiliations:** Department of Plant Cytology and Embryology, Faculty of Biology, Institute of Botany, Jagiellonian University in Kraków, 9 Gronostajowa St., 30-387 Kraków, Poland; bartosz.plachno@uj.edu.pl; Tel.: +48-12-664-60-39

Carnivorous plants (approximately 850 species) are specific mixotrophic plants which all perform photosynthesis but need mainly nitrogen and phosphorous from animal or protist bodies [1,2,3,4,5,6]. However, carnivorous plants are not only simple predators, their traps are also a specific home and habitat for many various mutualist organisms, some of which are even vertebrates [7,8,9,10,11,12,13,14,15,16,17,18]. It should be noted that even Charles Darwin was fascinated by these plants [19,20], which he called insectivorous in his book [21]. Interest in this ecological group of plants has been growing, especially as new techniques are emerging that offer new opportunities in research. Carnivorous plants are used as excellent models for cytological, genomic, and physiological studies, e.g., Professor Irene Lichtscheidl’s team was focused on the uptake of extracellular substances using endocytosis by the glands of carnivorous plants [22,23,24]. Freeze substitution, 3D electron tomography, and serial block face SEM provided new opportunities to understand the architecture of carnivorous plant cells [23,25,26,27]. The importance of jasmonate signaling in the functioning of traps in the case of prey recognizing and starting the digestion cycle has been studied [28,29,30]. Another important trend is the study of the genomes of carnivorous plants and genes related to carnivory and trap formation, e.g., [31,32,33,34,35,36].

This new Special Issue entitled “Carnivorous Plant Biology: From Gene to Traps” of the International Journal of Molecular Sciences includes a total of six contributions: five original articles and one review providing new information about the various aspects of carnivorous plant biology, containing phytochemistry, physiology, cytology, and ecology.

Miclea [37] extensively reviewed the recent progress in the field of secondary metabolites with significant biological activity found in the Sarraceniaceae family. She proved that *Sarracenia* is the most studied genus of the family in the case of metabolites. This review showed that secondary metabolites with significant biological activities, which can be isolated from *Darlingtonia*, *Heliamphora*, and *Sarracenia* plants, may be employed to promote human and animal health.

Kruppert et al. [38] investigated the interaction between an aquatic carnivorous plant bladderwort (*Utricularia* x *neglecta* Lehm, Lentibulariaceae) and *Ceriodaphnia dubia* (Crustaceans). This is a very innovative study because the authors found the inducible defenses of an animal against a coexisting plant predator in *C. dubia*. When crustaceans are cultivated with *Utricularia*, they show changes in morphology, life history, and behavior. This work is an important step for understanding how carnivorous plants influence not only animal behavior but even animal evolution.

The next two studies deal with carnivorous plants from the genus *Byblis*. Both papers are important because the trapping system of *Byblis* is poorly studied compared to other genera of carnivorous plants. Poppinga et al. [39] conducted a series of experiments using stimulation, kinematics, actuation, and light microscopy to study the stalked trichomes of *Byblis gigantea*. They proposed that the chemonastic movements of stalked trichomes may help *Byblis* to retain and digest its prey. Li et al. [40] used another *Byblis* species, *B. guehoi*, to test the theory that different leaf trichomes (stalked trichomes and sessile trichomes) play different roles in carnivory. They fed *B. guehoi* using fluorescein isothiocyanate-labeled bovine serum albumin to monitor the transport of nutrients. The authors showed that sessile trichomes play crucial roles in nutrient absorption by secreting various digestive enzymes and by absorbing large protein molecules efficiently.

Banaś et al. [41] studied the individual architectures and the values of the photosynthetic efficiencies of three sundew species (*D. anglica*, *D. intermedia*, and *Drosera rotundifolia*) and also identified the features of the abiotic environment that affect them. They found differences among the species: *D. anglica*, due to its very low photosynthetic efficiency, avoids competition by occupying highly hydrated habitats, while *D. intermedia* has adapted to occupy highly variable habitats in terms of hydration. According to the authors, the last species *D. rotundifolia* is best adapted to different environmental conditions, especially to variable light conditions. The results obtained will be extremely helpful in developing strategies for the active protection of these species.

Lustofin et al. [42] studied the morphology of Central American and Mexican *Pinguicula* species. These authors wanted to verify the two hypotheses: that both the distribution and diversity of non-glandular and glandular trichomes are connected to a type of pollinator, and that the distribution and diversity of non-glandular and glandular trichomes are more closely connected with the phylogenetical position. According to these authors, the flower morphology of the *Pinguicula* species with psychophily and ornithophily syndromes is similar, in contrast to the morphology of species with myophily/mellitophily syndrome. This is most probably as a result of adaption to a principal pollinator. They proposed that most micromorphological floral traits are potentially related to pollination syndromes, whereas only a small number of characteristics are shared among all species of *Pinguicula*.
